# Healthcare Professionals’ Perspectives on the Direct Referral Pathway for Syphilis Management in Western Sydney

**DOI:** 10.1007/s10508-026-03497-z

**Published:** 2026-08-13

**Authors:** Matthew N. Berger, Sophie Norton, Jason Ma, Jennifer Lampard, Conrad Moreira, Rohan I. Bopage

**Affiliations:** 1 Children’s Hospital Westmead Clinical School, Faculty of Medicine and Health, The University of Sydney, Westmead, NSW Australia; 2 Centre for Population Health, Western Sydney Local Health District, North Parramatta, NSW Australia; 3 Sydney Infectious Diseases Institute, Faculty of Medicine and Health, The University of Sydney, Camperdown, NSW Australia; 4 Office of the Chief Medical Health Officer, Vancouver Coastal Health, 800-601 W Broadway, Vancouver, BC V5Z 4C2 Canada; 5 Faculty of Science, The University of British Columbia, Vancouver, BC Canada; 6 Western Sydney Sexual Health Centre, Western Sydney Local Health District, North Parramatta, NSW Australia; 7 Westmead Clinical School, Faculty of Medicine and Health, The University of Sydney, Westmead, NSW Australia

**Keywords:** Syphilis, Syphilis in pregnancy, Sexual health, Pregnancy, Public health

## Abstract

Syphilis remains a major public health concern globally, with rising incidence contributing to preventable complications, including congenital syphilis. As such, the Western Sydney Local Health District implemented a direct syphilis referral pathway between the public health unit and sexual health services. This study aimed to explore healthcare professionals’ experiences of this direct referral pathway and its perceived impact on clinical outcomes and care coordination. A qualitative study was conducted as part of a quality improvement initiative. Semi-structured interviews were undertaken with clinicians from public health and sexual health services involved in the referral pathway. Interviews were audio recorded, transcribed verbatim, and analyzed using inductive and deductive thematic analysis. Fourteen clinicians participated, including medical practitioners and registered nurses. Two overarching themes were identified: (1) proactive case follow-up as a safeguard against preventable consequences of syphilis and (2) infrastructure and interprofessional relationships as enablers of sustained coordination. Participants reported that the pathway enabled proactive engagement of high-risk populations (i.e., pregnant women), resulting in faster treatment and reduced loss to follow-up. Improved interprofessional communication, clearer roles, and access to digital tools (e.g., shared referral forms and electronic medical records) enhanced coordination. Persistent challenges included stigma, unstable housing, and difficulties engaging some patients despite repeated outreach. Clinicians perceived the direct syphilis referral pathway as improving timeliness, coordination, and equity of care for high-risk patients. Strengthened collaboration between public health and sexual health services supported integrated management, although ongoing social and structural barriers highlight the need for complementary supportive interventions.

**Supplementary Information:**

The online version contains supplementary material available at https://doi.org/10.1007/s10508-026-03497-z.

## Introduction

Syphilis is a common sexually transmitted infection (STI) caused by the bacterium *Treponema pallidum*. Despite advancements in medical understanding and treatment methods, syphilis remains a global health challenge attributed to factors like stigma, limited healthcare access, and risky sexual behaviors (Workowski et al., 2021). Incidence rates of syphilis cases have been increasing globally (Tao et al., 2023). Vulnerable populations for syphilis infection include men who have sex with men (MSM), pregnant women, people living with human immunodeficiency virus (HIV), sex workers, and people experiencing homelessness and substance use (Tsuboi et al., 2021; Workowski et al., 2021). There are four stages of syphilis, including primary, secondary, latent, and tertiary (Nyatsanza & Tipple, 2016; Workowski et al., 2021). Neurosyphilis, ocular syphilis, or otosyphilis can occur at any stage (Workowski et al., 2021). Pregnant women with syphilis can vertically transmit during pregnancy, labor or birth to their child, causing congenital syphilis. Barriers to syphilis screening in pregnancy include geographical location, cultural or social influences, domestic violence, negative healthcare professional attitudes, and knowledge (Díaz-olavarrieta et al., 2007; Zhang et al., 2022). A review found that untreated maternal syphilis resulted in a 21% increase of stillbirth and fetal loss, 9.3% in neonatal deaths, and 5.8% in low birth weight/prematurity compared to women without syphilis (Gomez et al., 2013). Congenital syphilis morbidity and mortality rates are high, with an estimated 700,000 congenital syphilis cases globally in 2022 (World Health Organization, n.d.).

In Australia, infectious syphilis notifications have increased markedly over the past decade, rising by 85% from 11.9 to 22.1 per 100,000 between 2015 and 2024, with 5866 cases reported nationally in 2024 (79% male) (King et al., 2025). Although cases remain concentrated among males, rates among women aged 15–44 years have more than tripled, increasing the risk of syphilis in pregnancy and congenital infection. In New South Wales (NSW), 1840 infectious cases (90.5% male) and three congenital syphilis cases were reported in 2024, with 19 congenital cases notified since 2017; rates were highest in metropolitan Sydney (King et al., 2025; NSW Health, 2024). In metropolitan Sydney, infectious syphilis notifications are concentrated among gay, bisexual, and other men who have sex with men, while nationally, Aboriginal and Torres Strait Islander people experience disproportionately higher rates of infectious and congenital syphilis, reflecting broader inequities and disproportionate barriers experienced by Aboriginal and Torres Strait Islander peoples and women facing geographic, cultural, and social disadvantage (Hengel et al., 2024; King et al., 2025; NSW Health, 2024). Syphilis was declared a communicable disease incident of national significance by the Australian chief medical officer as of mid-2025 (Australian Centre for Disease Control, 2025). Public health intervention is key to reducing the number of untreated syphilis cases in the community, thus preventing transmission and reducing complications such as congenital syphilis (Peterman & Furness, 2015; Workowski et al., 2021). The NSW Health STIs Strategy 2022–2026 sets several key targets for syphilis control, including a 5% reduction in infectious syphilis notification rates by 2026; universal antenatal screening, with 100% of pregnancies tested at least once; the elimination of congenital syphilis; and ensuring that 95% of people diagnosed with infectious syphilis commence treatment within two weeks of testing (NSW Health, 2022).

There is limited research on direct referral pathways for high-risk or complex syphilis cases. Guidelines emphasize rapid follow-up and partner management, yet most studies assess isolated components rather than whole referral pathways. Despite the use of structured referral processes, there is limited empirical evidence on how integrated public health clinical pathways function in routine practice or influence accountability and care coordination. Addressing this gap is important in the context of rising syphilis incidence, where timely linkage, treatment verification, and continuity of care directly affect preventable outcomes such as congenital syphilis (Cope et al., 2026; Gratrix et al., 2022; Public Health Ontario, 2025). As such, this study aimed to explore the experiences and perspectives of healthcare professionals involved in the direct syphilis referral pathway between public health and sexual health services, with the focus on identifying barriers and facilitators in sustaining this intervention. We also explored the perceived clinical management and timeliness of complex syphilis cases through the referral pathway, with a view to understanding how these processes might be strengthened to support better patient care.

## Method

This qualitative study, conducted as part of a quality improvement project, explored the perceptions and experiences of clinicians involved in the syphilis referral pathway within the Western Sydney Local Health District (WSLHD). The Standards for Reporting Qualitative Research was used to guide the reporting of the study (O’Brien et al., 2014).

The direct syphilis referral pathway was an established framework implemented in 2019 within WSLHD prior to the commencement of this study. This intervention was developed by senior medical, nursing, and allied health staff (i.e., social workers, psychologists, and counselors) across Western Sydney Public Health Units (PHU) and Sexual Health Center (SHC). It was implemented due to rising syphilis cases and to prevent cases being lost to follow-up, specifically syphilis in pregnancy and congenital syphilis, which was a noted concern with the previous method. This qualitative evaluation examined clinicians’ experiences of this pathway as part of ongoing quality improvement efforts. The traditional referral process involved general practitioners (GPs) and other clinicians in the community sending referrals to specialist services for managing people diagnosed with syphilis. The direct syphilis referral pathway involves PHU clinicians directly referring syphilis notifications received from laboratories to the sexual health specialists when the testing GP needed support with syphilis management or was not contactable. A formal standard operating procedure (SOP) was established between the PHU and SHC outlining the direct referral process and roles and responsibilities of the staff involved in managing the referrals. A workflow of the referral pathway is displayed in Supplementary File 1.

### Participants

A purposive convenience sample of clinicians from public health and sexual health services was invited to participate in an online semi-structured interview. Clinicians included, but were not limited to, medical practitioners, nurses, social workers, and counselors. Clinicians were eligible to participate if they had been involved in the direct referral pathway in Western Sydney. The research team sent invitations to subscribers via public health and sexual health work group emails, which were restricted to district-employed clinicians. Additionally, flyers were displayed within the PHU and SHC. The number of staff subscribed to each group email was unknown; however, 15 individuals expressed interest in participating in an interview, but one did not attend.

### Measures and Procedure

Semi-structured interviews were conducted via Microsoft Teams, recorded, and subsequently transcribed verbatim. Written consent was obtained prior to the interview. All study materials, including consent forms and interview data, were stored securely on an NSW Health drive. Demographic information was collected during the first phase of the interview. Interviews were conducted by the research team within WSLHD (MNB and SN), and the interviewers were not involved in the syphilis referral pathway process to avoid potential bias. Interviews took place from January to March 2025. The semi-structured interview guide was developed iteratively, informed by relevant literature on syphilis care and service integration and implementation framework, reviewed and refined by the research team in consultation with senior clinicians, and pilot tested internally for clarity and flow prior to data collection. The interview guide included key questions on participants’ roles within the syphilis referral pathway and elicited perceptions of the barriers and facilitators to its implementation, as well as how the pathway has influenced inter-service collaboration and clinical outcomes. Specific questions probed process gaps, resource needs, relational dynamics between public and sexual health services, and opportunities to strengthen efficiency, patient care, and system-level integration. The interview guide is available in Supplementary File 2.

### Analysis

The interviews were analyzed utilizing inductive and deductive thematic analysis (Braun & Clarke, 2006). Analysis followed Braun and Clarke’s thematic analysis approach, including data familiarization, systematic generation of initial codes, and progressive development and refinement of candidate themes. Data were coded using NVivo software (QSR International, n.d.) by two researchers MNB and JM to ensure consistency. Transcripts were read and re-read prior to coding to enhance immersion in the dataset. Coding was conducted line by line, and codes were refined iteratively through comparisons within and across transcripts. Coding inconsistencies were discussed among the authors and resolved through consensus to ensure a shared interpretation of the data. JM was supported and trained by MNB on qualitative thematic analysis. Upon data saturation, when no new codes emerged, researchers independently identified overarching themes and patterns. The accuracy of these findings was verified by researchers.

## Results

### Overview

The 14 participants who completed interviews were aged 38–63 years. Nine (64.3%) identified as female and five (35.7%) identified as male. Half of the participants were born in Australia. Most participants (*n* = 12, 85.7%) were highly educated, possessing a postgraduate degree. Seven participants were working as medical practitioners and seven as registered nurses. Participants listed multiple specialties (*n* = 19), with sexual health (*n* = 9, 47.4%) being the most selected, followed by public health (*n* = 5, 26.3%), and general practice or other (*n* = 5, 26.3%). The majority (*n* = 11, 78.6%) had been working in their profession for more than 10 years. Further information is illustrated in Table 1.

**Table 1:** Participant demographics

Trait	Value	Participants (*n* = 14) % (n)
Gender	Female	64.3 (9)
	Male	35.7 (5)
Age (years)	< 40	21.4 (3)
	40–50	35.7 (5)
	> 50	42.9 (6)
Country of birth	Australia	50.0 (7)
	Outside Australia	50.0 (7)
Aboriginal or Torres Strait Islander descent	Yes	7.1 (1)
	No	92.9 (13)
Education	Undergraduate degree	14.3 (2)
	Postgraduate degree	85.7 (12)
Profession	Medical Practitioner	50.0 (7)
	Registered Nurse*	50.0 (7)
Specialty area** (*n* = 19)	Public Health	26.3 (5)
	Sexual Health	47.4 (9)
	General Practice / Other***	26.3 (5)
Years of experience in profession	6–10 years	21.4 (3)
	> 10 years	78.6 (11)

^*^Includes nurse practitioners. **Some participants had multiple specialties. ***Other specialties included women’s health and mental health

**Fig. 1:**
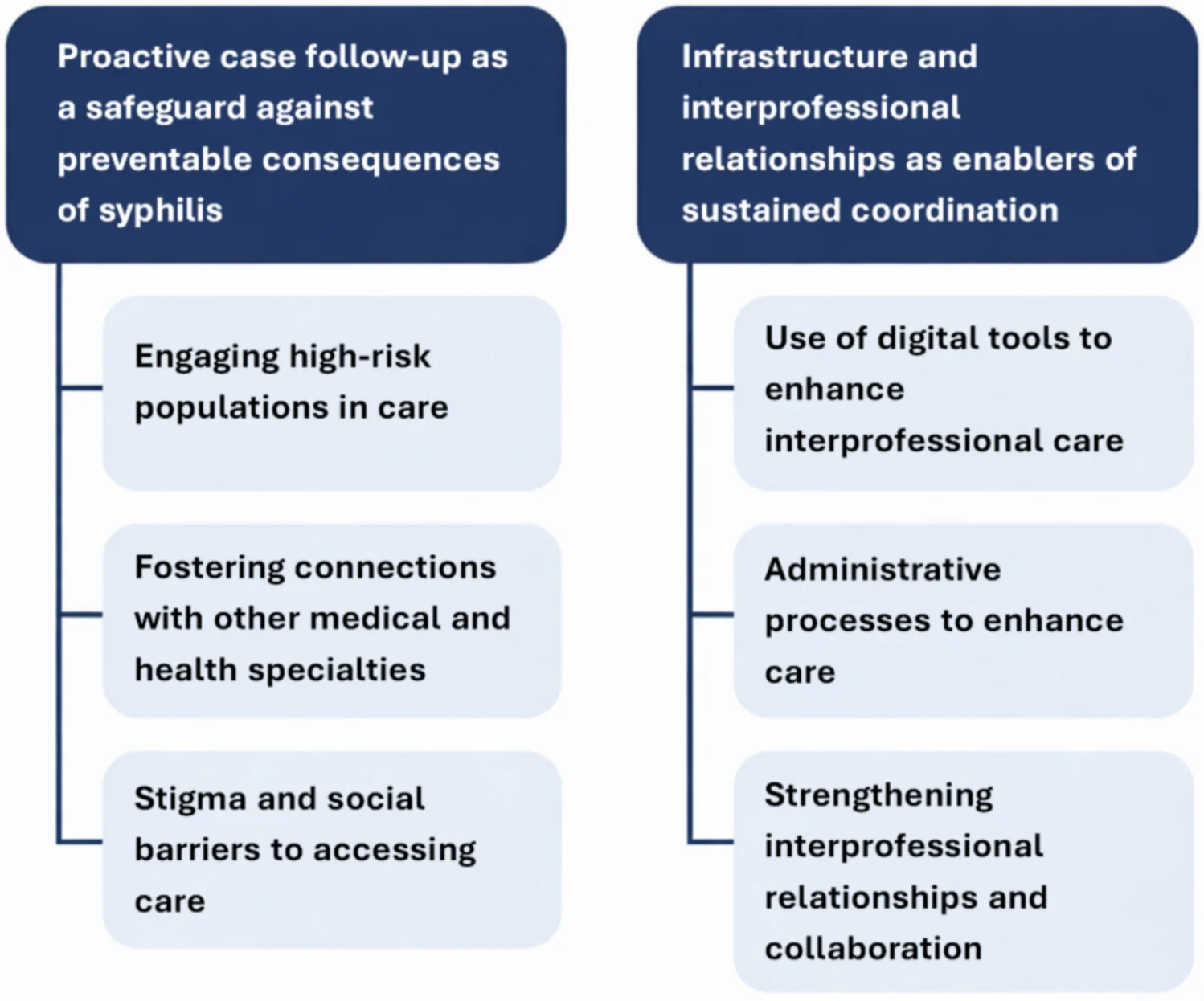
Thematic map of themes and subthemes

Two main themes were derived from the data: (1) proactive case follow-up as a safeguard against preventable consequences of syphilis and (2) infrastructure and interprofessional relationships as enablers of sustained coordination. Each theme consisted of three subthemes. This is displayed in Fig. 1. Quotes are presented along with participants’ pseudonyms, professions, and main specialty areas of practice.

### Proactive Case Follow-Up as a Safeguard Against Preventable Consequences of Syphilis

#### Engaging High-Risk Populations in Care

Participants emphasized that the syphilis referral pathway enabled proactive engagement of high‑risk clients, particularly those at risk of severe outcomes such as pregnant women with syphilis. In contrast with prior ad‑hoc approaches that relied on GPs to act on positive results, public health staff can now identify and directly refer priority patients to the SHC. Several interviewees perceived that the pathway reduced loss to follow-up, “minimizing the risk of any congenital syphilis” (Giovanni, medical practitioner, sexual health). Many clinicians believed that without the referral process, a considerable number of patients might not receive the care they need, leading to ongoing transmission or worse outcomes (e.g., congenital syphilis or neurosyphilis) with one stating that “these people probably would not have been treated if not for this pathway” and that it had “resulted in increased uptake of treatment and follow-up” (Matteo, medical practitioner, sexual health). Others observed that clients are seen and treated faster. Participants described the pathway as expanding the clinic’s reach beyond the traditional sexual health clientele, engaging people who previously would not have sought specialty services. Heterosexual men and women who test positive outside the sexual health service are now actively linked into care. Participants stressed the urgency of this proactive approach, noting that they often “only get one shot” to engage these patients before they disappear from the system.

[The case] would have just sat with a GP… and then if the GP couldn’t contact them, maybe just gone by the wayside. - Silvia, medical practitioner, sexual health

I think there’s lots of positive things about [the referral pathway] … we do prioritise … high urgency syphilis cases – that is, infectious syphilis in pregnant females and in newborns. - Enzo, medical practitioner, public health

### Fostering Connections with Other Medical and Health Specialties

Participants described greatly improved integration between the PHU and the SHC, as well as better coordination with GPs, antenatal and maternity services, and other relevant providers. Participants described clear communication pathways linking public health services with GPs and antenatal clinics. Knowledge sharing was also a benefit; by linking the PHU and sexual health clinicians, the pathway created opportunities to educate healthcare providers about syphilis management and the importance of prompt treatment and follow‑up. Participants referred socially complex cases to the SHC, as it also has access to social workers and interpreters. Through these links, patients dealing with domestic violence, homelessness, or language barriers can receive help from social workers, counselors, and interpreter services. Several participants mentioned that GPs appreciated having specialist input for managing syphilis, especially in unfamiliar or complex scenarios. The pathway offered clear avenues for GPs to hand over or co‑manage care with the SHC; “they’re not stuck with the patient [whom] they may not feel confident treating” (Sofia, registered nurse, public health). Participants perceived the referral pathway as building strong connections not only between the PHU and SHC, but also extending outward to engage GPs, maternity and antenatal services, and allied supports, ensuring that referred patients received comprehensive, coordinated care across medical specialties.

The GP education … it’s [syphilis] a very hard topic, you know, a hard health issue, not easy to stage. This report could help … more engagement and talks with the sexual health physicians. - Nico, medical practitioner, sexual health

The advantage has been that Western Sydney’s sexual health have social health services and they’re able to throw everything at the number of complexities. - Alessia, registered nurse, public health

### Stigma and Social Barriers to Accessing Care

Many referred patients were described as coming from vulnerable or marginalized groups with complex life circumstances that made engagement difficult. Clinicians noted that some patients live in unstable conditions and have mental health conditions, experiencing homelessness or substance use. Cases of infectious syphilis in pregnancy exemplified these challenges; some had multiple social risk factors such as domestic violence or homelessness. Social barriers meant that it could be “challenging for sexual health to contact them and [for] them to remain engaged in services” (Francesca, registered nurse, public health). Despite repeated contact attempts, some patients did not answer or failed to attend appointments. “Did not attend rates” (Silvia, medical practitioner, sexual health) were a persistent issue. Hesitancy was often attributed to fear, denial, or the stigma associated with having an STI and visiting a sexual health clinic. Participants agreed that stigma, whether anticipated or experienced, remains a barrier. Participants felt that the supportive, non‑judgmental approach of the referral pathway could help alleviate these issues.

I guess occasionally we pick up on say an MSM who isn’t engaged in appropriate care and whose GP perhaps didn’t even know that they were having sex with men ... let’s talk about PrEP [pre-exposure prophylaxis for HIV], mpox vaccines and all that kind of stuff. – Silvia, medical practitioner, sexual health

I have then created an mpox referral form ... because there seemed to be a large gap in GP managed mpox treatment and care as opposed to SHC treatment and care of mpox cases, there’s a lot of stigma. – Nico, medical practitioner, sexual health

### Infrastructure and Interprofessional Relationships as Enablers of Sustained Coordination

#### Use of Digital Tools to Enhance Interprofessional Care

Participants described digital tools as central to the syphilis referral pathway, enhancing the efficiency of interdisciplinary collaboration between the PHU, SHC, and primary care. The referral pathway form, a joint effort between PHU and SHC staff, was broadened to collect more detailed information, such as sex at birth and gender identity, and recently underwent a “re-jig … to do a quicker referral” (Lucia, registered nurse, public health) through drop-down menus and a fillable format, making it “an easy way to make that referral to sexual health, which isn’t labour intensive” (Francesca, registered nurses, public health). Supporting tools, including the Sexual Health Referral Tracker database and shared access to the sexual health electronic medical record (eMR), further strengthened interdepartmental coordination. The Referral Tracker allowed public health staff to view referred cases and monitor follow-up, while access to the eMR provided visibility into patient progress, including clinical notes and treatment updates.

The big thing that has really contributed to the success of how well this [referral pathway] works has been the access provided to communicable disease clinicians to the sexual health eMR. – Alessia, registered nurse, public health

### Administrative Processes to Enhance Care

Participants highlighted several administrative processes that underpin the efficiency and effectiveness of collaboration within the referral pathway. Participants reported that having preliminary access to information (e.g., previous test results and GP information) facilitated more timely planning. Clinicians emphasized that they could get patients “sorted out in that first day” (Silvia, medical practitioner, sexual health) rather than having to gather information later by calling patients back, risking them being lost to follow‑up. The SOP was described as succinct and easy to understand, providing clear guidance on the referral process and listing useful contacts. New staff members found the SOP especially helpful for ensuring that the pathway is undertaken consistently; “The SOP… not overly wordy and fairly easy to get your head around” (Martina, registered nurse, sexual health). Participants perceived that eMR access reduced the need for repetitive communication.

I can generally get them sorted out in that first day rather than having to try and get all the information from the patient. – Silvia, medical practitioner, sexual health

The SOP of the pathway [is] especially useful for new staff such as myself ... to ensure that the pathway is consistent and undertaken consistently. – Francesca, registered nurse, public health

### Strengthening Interprofessional Relationships and Collaboration

Participants described improved communication “correct information is being communicated across promptly and in a timely manner” (Sofia, registered nurse, public health). Public health and sexual health staff likened their relationship to that of siblings and expressed gratitude for the quality of communication; “I think there’s good personal relationships… I’ve often reached out directly … that’s great [to have] a single named person”—Martina, registered nurse, sexual health. Designated contact persons further strengthened collaboration, enabling rapid coordination and proactive communication, was perceived to reduce delays in timely follow‑up. Some participants noted that strengthened relationships were also helpful during other outbreaks, “we built up very strong rapport, which has helped with other outbreaks like the mpox and so forth” (Giovanni, medical practitioner, sexual health). Participants expressed concerns regarding sustainability as referral numbers rise.

That ability just to quickly send off an email or a Teams message... for everyone to actually know what they need to do and respond... so we all seem to be on the same page. – Isabella, registered nurse, sexual health

I think the relationship between public health and sexual health continue[s] to grow stronger and stronger. We’re doing a lot of collaboration with them, with different working groups, focusing on different areas of STIs. – Francesca, registered nurse, public health

## Discussion

The findings highlighted the pivotal role of the direct syphilis referral pathway between public health and sexual health in enabling timely, coordinated, and equitable syphilis care. The pathway expedited treatment, especially for urgent cases (i.e., pregnant women). It also fostered meaningful multidisciplinary collaboration among providers. Participants noted that while barriers remained (e.g., stigma, unstable housing, limited patient engagement), the referral pathway was crucial for identifying and addressing these challenges. By referring patients to care and social support, the pathway not only improved treatment uptake and follow-up but also extended services to populations previously underserved by sexual health services. Combatting rising syphilis infection rates requires combined efforts among public health and clinical services, along with community outreach and provider education (Singh, 2019). Our findings align with growing international evidence that fragmented systems contribute to missed or delayed syphilis treatment and that integrated public health responses are required to improve care continuity. This referral pathway mirrors recommendations for renewed focus across health systems to address syphilis, breaking down silos between disciplines (MacKenzie et al., 2023; Sawleshwarkar et al., 2026). Recent literature has highlighted that syphilis resurgence has led to increased cases of congenital syphilis and multiple infant deaths, underscoring the urgency for system-wide action (Flores et al., 2025; McDonald et al., 2023; Sawleshwarkar et al., 2026). In this light, our study’s pathway is a practical example of a coordinated response that experts advocate to address rising syphilis cases (Tetteh & Moore, 2025).

Participants described how linking medical management with psychosocial support helped engage marginalized patients who might otherwise be lost to follow-up. This is consistent with evidence demonstrating that structural vulnerabilities, including unstable housing, stigma, and limited healthcare access, are key barriers to syphilis treatment completion. This resonates with views that combatting syphilis requires holistic, patient-centered strategies that integrate social support (Tetteh & Moore, 2025). By prioritizing vulnerable individuals (including those with unstable living situations or who face stigma) and coordinating partner notification, the pathway addressed social determinants that often hinder sexual and reproductive healthcare (Moore et al., 2023; Traeger & Stoové, 2022). Referral pathways have been seen in other reportable communicable diseases, such as HIV, promoting efficient engagement in care (Philbin et al., 2016). External analyses emphasize that purely clinical solutions are insufficient; effective syphilis control must involve intersectional, collaborative strategies targeting structural barriers to health equity (Tetteh & Moore, 2025). For example, offering culturally safe, stigma-free sexual health services can empower patients to seek testing and treatment earlier (Tetteh & Moore, 2025). In many high-income settings, syphilis referral pathways rely on antenatal screening teams referring positive cases to specialist services, public health-led follow-up of notifications, or multi-service coordination without a single referral node, often supported variably by electronic reporting systems (NHS England, 2025; Paneth-Pollak et al., 2010; Public Health Ontario, 2025). The Western Sydney referral pathway bridged public health and sexual health services through a direct, PHU-initiated referral from laboratory notifications, with defined triage timeframes and shared digital tools to support treatment verification and follow-up, illustrating how interprofessional practice may improve syphilis case management.

Public health programs could adapt similar referral systems to ensure timely treatment of infectious syphilis cases, particularly high-priority cases, thereby reducing community transmission and preventing complications such as congenital syphilis. A recent quality improvement project in Chicago, USA, demonstrated increased awareness of congenital syphilis cases through collaboration between pediatric infectious diseases and the local PHU (Flores et al., 2025). Incorporating training on the referral pathway into continuing education for GPs and prenatal care providers could further increase its utilization and effectiveness (Montag et al., 2024; Wagman et al., 2022). A recent Chicago, US initiative introducing opt-out syphilis screening in emergency departments increased detection of asymptomatic cases, including among pregnant women (Stanford et al., 2024). There is a need to expand screening, particularly for heterosexual women, as part of routine care across health services (i.e., routine STI screening, prenatal care, drug and substance use services, and community-led providers) (Carter et al., 2023; Kimball et al., 2020; Tucker et al., 2010). This referral pathway could be expanded to other infections, such as mpox, where MSM could be linked to specialist sexual health services to reduce potential stigma (Berger et al., 2025). Future studies should investigate whether these systems shorten time to treatment, increase treatment completion rates, or reduce rates of syphilis sequelae (e.g., neurosyphilis or congenital syphilis). In the context of rising syphilis rates, public health services should consider strengthening and expanding existing approaches to case management and prevention. Nonetheless, implementation must be assessed within local service contexts, with consideration of feasibility and effectiveness, including cost-effectiveness, workforce capacity, time to treatment, and medication supply chains, particularly the availability of benzathine penicillin, given documented global shortages (Nurse-Findlay et al., 2017).

### Limitations

This study has several limitations. Perspectives were limited to public health and sexual health clinicians, and patient experiences were not captured. The study was conducted within a single local health district with a small sample, limiting generalizability. Participation was voluntary, introducing potential self-selection bias, and findings reflect participant perceptions, which may be subject to recall or social desirability bias.

### Conclusion

This study explored the experiences and perspectives of healthcare professionals involved in the direct syphilis referral pathway between the Western Sydney PHU and SHC, with particular attention to barriers, facilitators, and perceived effects on clinical care and timeliness for complex cases. Overall, the pathway was perceived as strengthening coordination, expediting treatment, and improving follow-up for high-priority patients. By formally linking surveillance with specialist care, it addressed system inefficiencies and reduced barriers such as stigma and fragmented communication. These findings highlight the value of integrated, interprofessional models in supporting syphilis control and congenital syphilis prevention. Future research should evaluate clinical outcomes, scalability, cost-effectiveness, and patient experiences to inform broader implementation of streamlined referral systems in STI and communicable disease management.

## Supplementary Information

Below is the link to the electronic supplementary material.

Supplementary file1 (PDF 275 KB)

Supplementary file2 (PDF 62 KB)
