# Supplementary material for: Healthcare Professionals’ Perspectives on the Direct Referral Pathway for Syphilis Management in Western Sydney
Source: Arch Sex Behav. 2026 Aug 13;55(6):2679–87. doi: 10.1007/s10508-026-03497-z (PMC13597549; doi:10.1007/s10508-026-03497-z)
Supplement: Supplementary file 1 [file 10508_2026_3497_MOESM1_ESM.pdf]

Western Sydney Direct Syphilis Referral Pathway Workflow

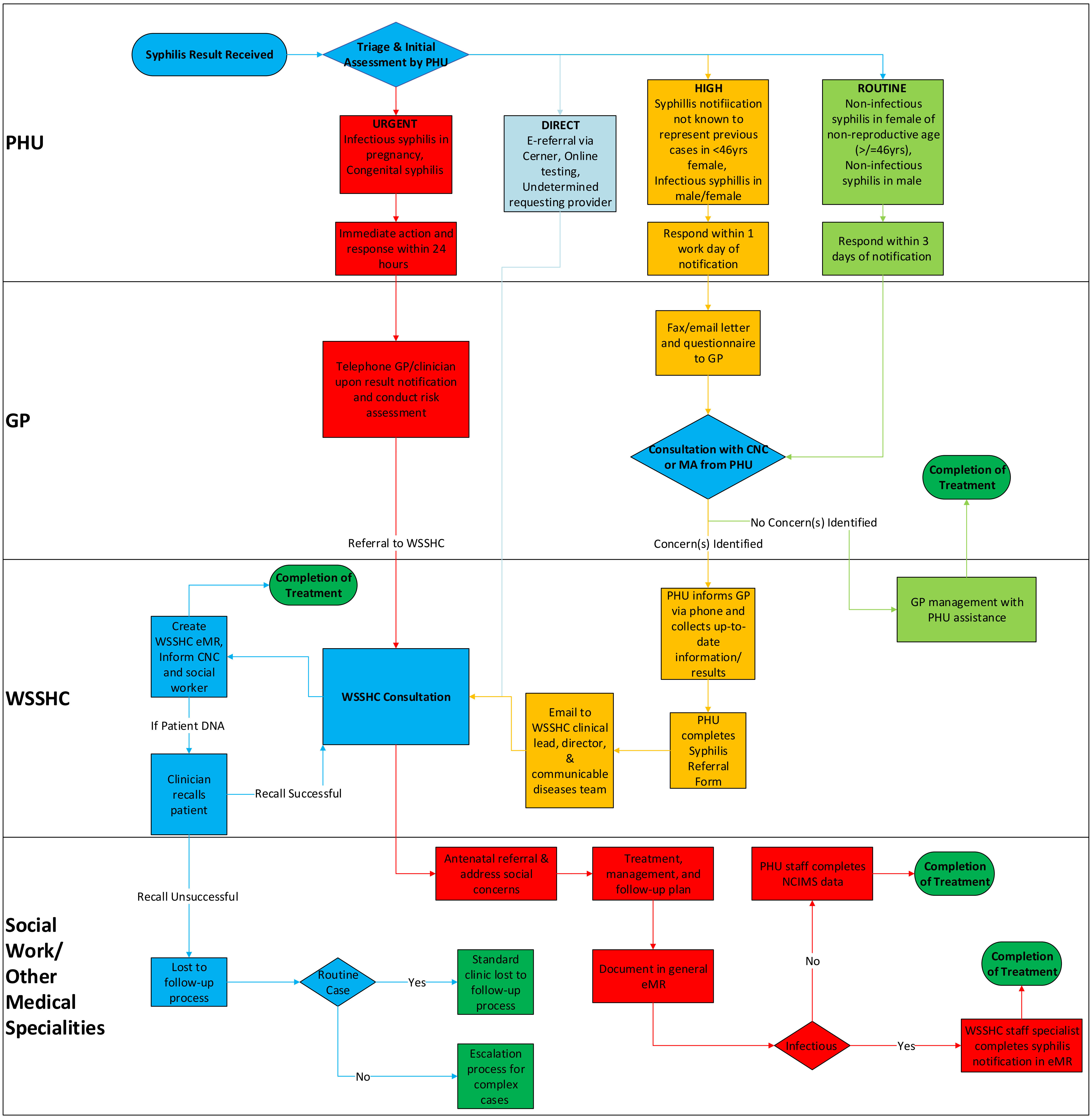

Abbreviations: CNC, Clinical Nurse Consultant; eMR, Electronic Medical Record; DNA, Did Not Attend; GP, General Practitioner; MA: Medical Advisor (Public Health Physician); NCIMS: Notifiable Conditions Information Management System; PHU, Public Health Unit; WSSHC, Western Sydney Sexual Health Centre.
