# Supplementary material for: Healthcare Professionals’ Perspectives on the Direct Referral Pathway for Syphilis Management in Western Sydney
Source: Arch Sex Behav. 2026 Aug 13;55(6):2679–87. doi: 10.1007/s10508-026-03497-z (PMC13597549; doi:10.1007/s10508-026-03497-z)
Supplement: Supplementary file 2 [file 10508_2026_3497_MOESM2_ESM.pdf]

Supplementary File 2: Interview guide

1. Could you please describe your role in the syphilis referral pathway?
   a. (Prompt: what is your involvement in syphilis care and management?)

2. Are you aware that there is a formalised agreement for the referral pathway of syphilis clients between Western Sydney Public Health Unit and Sexual Health Centre?

3. What do you feel works well in the referral pathway?
   a. (Prompt: do you feel there are any positive outcomes for the client compared to routine care? (e.g., care in a GP clinic))

4. What do you feel does not work well in the referral pathway?
   a. (Prompt: do you feel there are any negative outcomes for the client compared to routine care?)

5. Are there any improvements that could be made?

6. How has yours or your team's role or processes changed over time (since the referral pathway was implemented)?
   a. (Prompt: Have you noticed any changes to your practice since working in this referral pathway before it was implemented in 2019 or to your other/previous role?)

7. Can you describe the relationship between public health and sexual health, and has this pathway made changes?
   a. (Prompt: the impact this pathway has had on that relationship)

8. Relating to the parts of the referral pathway that are relevant to you, has there been changes made to the referral pathway?
   a. If not, which parts have been removed or added and why?
   b. Have extra resources been developed to assist the process?

9. Do you feel that this referral pathway has an impact on clinical outcomes for the patient? (Prompt: have you recognised any complications that have been flagged? E.g., domestic violence, pregnancy or any social issues.)

10. Have you recognised any common issues that should be addressed?

a. (If so, what do you think should be done to address the issue?)

11. How do you think the process can be improved?

a. (Prompt: What other resources are needed to improve or expand the process?)

12. Do you have any other comments you would like to make about syphilis or the referral pathway?
